# Moving from Virtual Reality Exposure-Based Therapy to Augmented Reality Exposure-Based Therapy: A Review

**DOI:** 10.3389/fnhum.2014.00112

**Published:** 2014-03-04

**Authors:** Oliver Baus, Stéphane Bouchard

**Affiliations:** ^1^School of Psychology, University of Ottawa, Ottawa, ON, Canada; ^2^Department of Psychoeducation and Psychology, Université du Québec en Outaouais, Gatineau, QC, Canada

**Keywords:** virtual reality, augmented reality, phobia, exposure therapy, synthetic environments

## Abstract

This paper reviews the move from virtual reality exposure-based therapy to augmented reality exposure-based therapy (ARET). Unlike virtual reality (VR), which entails a complete virtual environment (VE), augmented reality (AR) limits itself to producing certain virtual elements to then merge them into the view of the physical world. Although, the general public may only have become aware of AR in the last few years, AR type applications have been around since beginning of the twentieth century. Since, then, technological developments have enabled an ever increasing level of seamless integration of virtual and physical elements into one view. Like VR, AR allows the exposure to stimuli which, due to various reasons, may not be suitable for real-life scenarios. As such, AR has proven itself to be a medium through which individuals suffering from specific phobia can be exposed “safely” to the object(s) of their fear, without the costs associated with programing complete VEs. Thus, ARET can offer an efficacious alternative to some less advantageous exposure-based therapies. Above and beyond presenting what has been accomplished in ARET, this paper covers some less well-known aspects of the history of AR, raises some ARET related issues, and proposes potential avenues to be followed. These include the type of measures to be used to qualify the user’s experience in an augmented reality environment, the exclusion of certain AR-type functionalities from the definition of AR, as well as the potential use of ARET to treat non-small animal phobias, such as social phobia.

According to Moore’s law, the number of transistors on integrated circuits doubles approximately every 2 years (Moore, 1965). This growth leads to an exponential growth of technological capabilities. Innovative minds are applying the potential of these new technologies in what, historically, may have been technology aversive fields; mental health was one of those fields. Today, however, it is widely recognized that new technologies such as virtual and augmented realities are showing strong potential in that same field, and more specifically, in the treatment of phobia (Wrzesien et al., 2011a).

The objective of this paper is twofold. First, it reviews the move from virtual reality (VR) systems to augmented reality (AR) systems in the treatment of phobias. Second, it highlights four issues relating to AR: (a) qualifying an AR experience necessitates a set of AR specific instruments [not necessarily those used to qualify a virtual environment (VE) experience]; (b) historically, AR applications have been around a long time before the term “AR” was assigned to the concept; (c) presently, certain AR-type functionalities are excluded from the definition of AR; and (d) the use of augmented reality exposure-based therapy (ARET) has advantages over virtual reality exposure-based therapy (VRET), but these advantages could be exploited beyond the treatment of small animal phobia.

To this aim, the article first addresses some of the evolutions that have led to the use of AR in the treatment of specific phobias. To establish the framework of AR, it is useful to distinguish it from VR. Thus, the paper presents some definitions relating to the technology (VE, VR, and immersion), some of the concepts commonly used to quantify and qualify a user’s experience of virtual stimuli (presence, realism, and reality), as well as some of the non-mental health applications of VR. After having covered these basics, the focus shifts toward mental health. More specifically, the implications of suffering from a phobia and two of the possible (traditional) treatments, *in imago* and *in vivo* exposure-based therapies, are presented. Next, VRET, the “direct ancestor” to ARET, is introduced; its documented successes, as well some of as its advantages over traditional exposure-based methods are presented. After this overview, the focus shifts toward AR, including how it distinguishes itself from VR, some of its advantages over VR, and what criteria must be met to consider a functionality as AR. At this point, the instruments presently used to measure an AR user’s experience are discussed, and some concepts to be measured in AR are proposed. The next section addresses the history of AR. This is accomplished in two parts. While the first covers, some of the major events that occurred after the coining of the phrase “augmented reality,” the second addresses the period going back to the roots of AR, a time-frame less covered by previous publications. From this historic account will emerge an issue relating to the definition of AR: the present one leaves certain AR-type functionalities nameless. A solution will be proposed to close this semantic gap. Next, the AR enabling technologies, and some of the technical challenges faced by the developers are put forward. A variety of AR applications are listed. In particular, publications pertaining to the use of ARET are reviewed; these eight studies either test the efficacy of ARET protocols, compare ARET protocols to other types of exposure-based protocols, compare ARET technologies, or simply quantify users’ experiences in an ARET environment. Finally, the last discussion point addresses the limited use of ARET in the treatment of phobias, other than small animal phobias. One of the plausible reasons behind this self-imposed restriction, a possible way to break free of it, as well as the potentially resulting opportunity to expand ARET to the treatment of social phobia are discussed. To close, a conclusion reiterates the major points of the paper.

## Virtual Reality

### Virtuality and associated concepts

#### Virtual environment

The exact definition of the word “virtuality” depends on the context of its use. However, in the domain of VEs, Theodore Nelson’s definition is pertinent; he defines the virtuality of a thing as the “seeming” of that thing (Skagestad, 1998). Indeed, a VE consists of objects or entities seemingly “real” because they share at least one attribute of the “real thing” (usually the appearance), without sharing all of its physical characteristics (volume, weight, surface friction, etc.).

**Figure 1:**
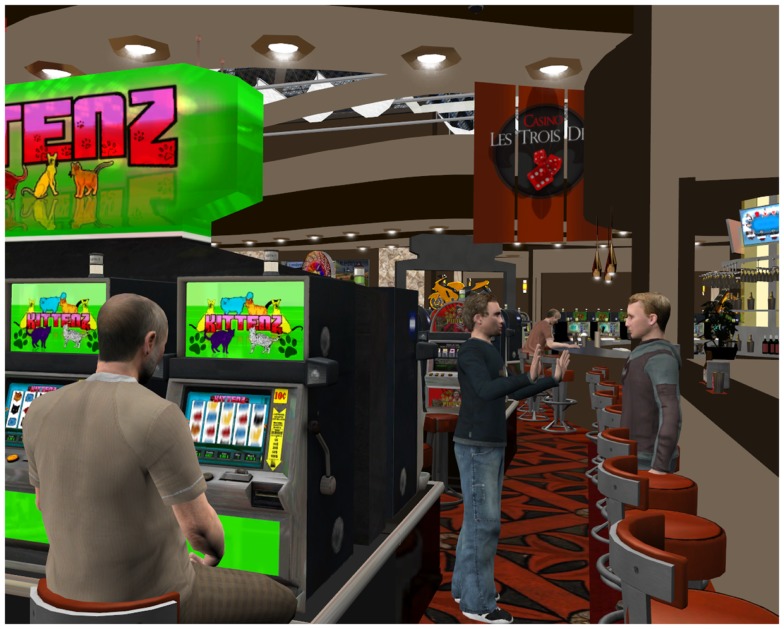
**Example of a virtual environment**. Credit, Laboratory of Cyberpsychology, Université du Québec en Outaouais.

A VE can be defined as a 3D digital space generated by computing technology (e.g., the scenario of a video game). It is comprised of visual stimuli projected on a surface (e.g., a wall, a computer screen, screens of a head mounted display) and, generally, acoustic stimuli produced by an electronic device (e.g., a headset, speakers). Further to these, a VE may also expose the user to haptic (contact), olfactory, or even gustatory stimuli (Sundgren et al., 1992; Burdea and Coiffet, 1994; Kalawsky, 2000; Fuchs et al., 2006). A VE aims to “extract” the user from the “physical” world and “insert” him into a synthetic world; this is accomplished by exposing him to synthetic sensory information that emulates real life stimuli (see Figure 1).

#### Virtual reality

Virtual reality is an application that, in very near real time, allows a user to navigate through, and interact with, a VE (Pratt et al., 1995). Depending on the type of system and programing, the user may interact with the environment from an egocentric point of view (also known as “first person point of view”) or an allocentric point of view (also known as “third person point of view”); in the case of the latter, the user moves a virtual representation of himself (called “avatar”). The user may also act upon virtual objects, and even interact with virtual beings (e.g., persons, animals). Compared to more passive media such as radio and television, the higher levels of cognitive, social, and physical interactivities of VR can boost the effect of the VE on the user (Fox et al., 2009). In more immersive egocentric VR systems, the user can interact with the VE via his own movements by wearing at least one input device (known as “tracker”). The latter detects its own position in space and transmits it continuously to a computer which: (a) continuously compares this data with the database associated to the VE; (b) determines the synthetic stimuli to be triggered; and (c) triggers the output devices to deploy them. All of this is accomplished in very near real time and can implicate multiple sensory modalities. Generally, worn at head level, a tracker may support three degrees of freedom (3-DOF) or six degrees of freedom (6-DOF) tracking; while a 3-DOF tracker allows tracking of head rotation only, the 6-DOF version tracks head rotation as well as horizontal and vertical displacements. Trackers may also be used to track specific body parts (e.g., the hands). Generally, the user of a 6-DOF system uses own body movement for small positional adjustments (turning around, bending down, repositioning, etc.) and a hand-held device (e.g., joy-stick, space ball, 3D mouse) to move over greater distances, such as walking from room to room in an apartment.

#### Immersion

**Figure 2:**
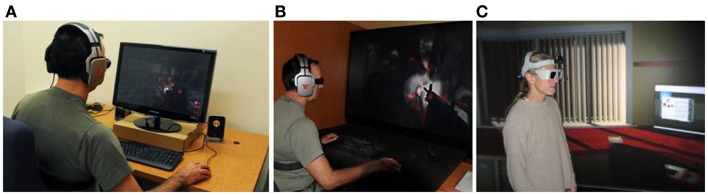
**Examples of immersion levels: (A) a non-immersive VR system, (B) a semi-immersive VR system, and (C) an immersive VR system**. Credits: **(A,B)** Bouchard et al. (2012a) by (SAGE Publications) reprinted by permission of SAGE **(C)** Laboratory of Cyberpsychology, Université du Québec en Outaouais.

Factors such as the number of senses stimulated, the number of and the level of interactions, as well as the fidelity of the synthetic stimuli contribute to a VR system’s level of immersion (Slater et al., 2009). This concept corresponds to the quality and the quantity of the stimuli employed to simulate the environment; it is an objective characterization of the system (Sanchez-Vives and Slater, 2005). At the same time, the level of immersion is also dependent on the ability of the system to isolate the user from stimuli foreign to the VE (e.g., room lights and external noise). Ma and Zheng (2011) use the following guidelines to distinguish between non-immersive, semi-immersive, and immersive VR systems: a non-immersive VR system employs conventional graphics workstation with a monitor, a keyboard and a mouse; a semi-immersive system uses a relatively high performance graphics computing system coupled with a large surface to display the visual scene; and an immersive VR system projects the visual scene into some kind of head mounted device – or large projection surfaces “encasing” the user – completely filling the user’s field of view (see Figure 2). The level of immersion, in turn, affects the user’s experience in the VE. Three of the dominant concepts used to measure the quality of the user’s experience are: the feeling of presence, the level of realism, and the degree of reality.

#### Presence, realism, and reality

While various definitions of presence have been proposed, Heeter (1992) views presence as a complex feeling composed of three dimensions: (a) personal presence refers to the feeling of actually being in the VE (versus in the physical room where the immersion takes place); (b) environmental presence refers to the feeling that the VE seems to acknowledge the user by reacting to his actions; and (c) social presence refers to the feeling of not being alone in the VE (Heeter, 1992). Compared to other definitions, the strengths of this one include its fidelity to the actual term “presence,” its simplicity, as well as its ability to account for the interactions between the user and the virtual location, objects, and animated entities; thus, Heeter’s conceptualization of presence will serve as reference to this article. The concept of presence, however, is not unique to VEs: watching a movie, a play or a painting, as well as reading a text or listening to the radio can induce a feeling of presence (Nash et al., 2000).

The level of realism corresponds to the degree of convergence between the expectations of the user and the actual experience in the VE (Baños et al., 2000). Thus, a virtual stimulus that meets the expectations of the user, such as an orange that smells like an orange, is likely to be rated as more realistic as that same orange if it smelled like nothing at all, or if it smelled like fish.

The level of reality refers to the level by which the user experiences the immersion as authentic (Baños et al., 2000). It is felt in response to the stimuli. Thus, a higher level of realism should be associated with a higher level of reality.

### VR applications

Today, the fields in which VR is used are numerous; they include education, health care, communication, engineering, and entertainment (Schuemie, 2003). Within these fields, VR applications may be used for a variety of purposes including pain management (Gold et al., 2007; Hoffman et al., 2008), virtual “visits” of construction projects in development (Brooks et al., 1992), the development of virtual classrooms (Moreno and Mayer, 2007), and collaborative work environments in which the users interact via avatars (Normand et al., 1999; Benford et al., 2001; Joslin et al., 2004; Reeves et al., 2008).

Often, VR is employed as a training tool. In such a function, its advantages include reduced cost, interactivity, and safety. Indeed, VR can offer financially advantageous active learning experiences involving scenarios that are too difficult and/or too dangerous to practice “real world.” Furthermore, its interactivity (Bailenson et al., 2005) as well as the possibility to pre-program a variety of training scenarios at multiple levels of difficulty can facilitate better learning. VR training applications include: visual inspections of aircraft with various structural flaws (e.g., Vora et al., 2002), the operation of various vehicles (e.g., Tichon et al., 2006), rapid and efficacious decision making by medical doctors (e.g., de Leo et al., 2003; Mantovani et al., 2003; Johnsen et al., 2006; Kenny et al., 2007) and by soldiers (e.g., Hill et al., 2003) in stressful situations, pre-deployment inter-cultural communication training prior to military deployments (e.g., Deaton et al., 2005), emergency management (e.g., Viciana-Abad et al., 2004), surgical procedures (e.g., O’Toole et al., 1998; Harders et al., 2008; Spitzer and Ackerman, 2008), rehabilitation (Rose et al., 1996; Jaffe et al., 2004; Crosbie et al., 2007), stress management (e.g., Bouchard et al., 2012b), and fear management training in the face of a phobia inducing stimulus (e.g., Côté and Bouchard, 2005). The latter form of “training” is more commonly known as VRET. Indeed, VRET is essentially a training activity during which an individual learns to master a task that he is incapable of carrying out: facing a particular stimulus without experiencing unwanted psychological and/or physiological reactions.

## Phobia

### Definition

While about 9% of the citizens of the United States were reported to suffer from a specific phobia (Gadermann et al., 2012), 60–80% of those affected have been reported not to seek treatment (Agras et al., 1969; Boyd et al., 1990; Magee et al., 1996; Essau et al., 2000). Suffering from a phobia means an individual experiences excessive anxiety when exposed to a certain stimulus; the trigger stimulus may be a specific entity (e.g., an animal species) or a situation (e.g., addressing a group of people, driving). In association to the elevated stress and anxiety, the individual may experience increased heartbeat, sweating, and dry mouth (Abate et al., 2011). In either case, the unrealistic and excessive fear of the stimulus can lead to avoidance behaviors that interfere with the subject’s life. Numerous studies suggest that exposure-based treatment is effective in treating phobic fear and avoidance behavior (e.g., Öst, 1989; Öst et al., 1991a, 1997). A lack of treatment can lead to a self-feeding spiral where increasing unrealistic fear feeds avoidance behaviors which, in turn, feed further fear. Untreated, this condition can lead to significant social and economic costs to society (Kessler and Greenberg, 2002; Kessler et al., 2008).

### Treatment of phobia

#### *In imago* and *in vivo* exposure-based therapies

Years of empirical work point to the efficacy of exposure-based therapy across a variety of anxiety disorders (Richard et al., 2007), and various theories have been proposed to explain its mechanisms of action. These include: the Two-Factor Theory of Fear Acquisition and Maintenance (Mowrer, 1960), the Bioinformational Theory (Lang, 1977), the Emotional Processing Theory (Rachman, 1980), the Emotional Processing Theory Model (Foa and Kozak, 1986), a revised version of the Emotional Processing Theory (Foa and McNally, 1996), the Perceived Control and Self-Efficacy Theory (Mineka and Thomas, 1999), as well as various Neural Networking Models (e.g., Tryon, 2005). Exposure-based treatments do not limit themselves to exposure sessions: the exposure is just the behavioral component of what usually amounts to a cognitive-behavioral protocol. Thus, an exposure-based treatment includes a broader set of behavioral and cognitive therapeutic techniques, including case formulation, cognitive restructuring, relapse prevention, etc.

The exposure component generally implies a gradual hierarchical exposure to the object of the fear in a safe and controlled way. The exposure aims to help the patient convincingly learn that the consequences he fears do not necessarily happen. According to the Emotional Processing Theory (Rachman, 1980; Foa and Kozak, 1986; Foa and McNally, 1996), the exposure works because it allows the patient to fully experience the activation and subsequent natural reduction of fear in presence of the phobia inducing stimulus (Abramowitz, 2013). Thus, the use of “crutches” (e.g., relaxation exercises) or downright avoidance behaviors (e.g., behaviorally or cognitively ignoring the stimulus) can be detrimental to the clinical efficacy of the exposure (Abramowitz, 2013). More recent models explaining the therapeutic mechanisms of exposure (e.g., Bouton and King, 1983; Craske et al., 2008) propose that the result of a successful exposure-based treatment is not the disappearance of the previously learned association between the stimulus and perceived threat, but the creation of a newly learned association that competes with the old dysfunctional one; repeated exposures, and non-avoidance behaviors are meant to establish, strengthen, and maintain the functional response such that it may “overpower” the dysfunctional response, and continue to do so in the long term.

**Figure 3:**
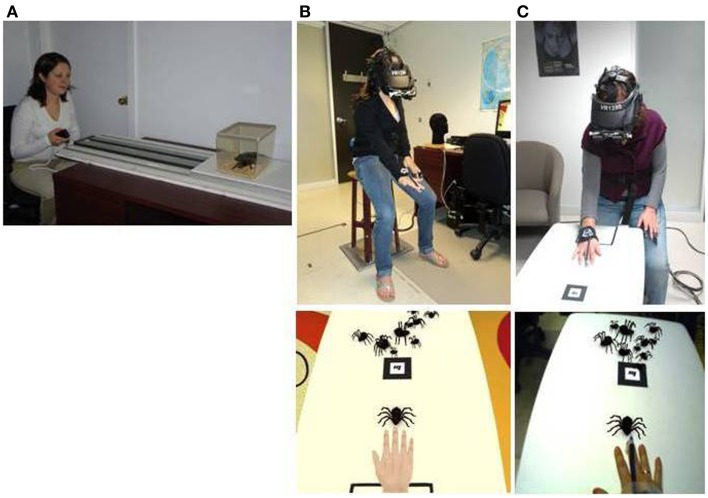
**Three types of exposure-based methods: (A) *in vivo* exposure, (B) virtual reality exposure (bottom photo shows the user’s view), and (C) augmented reality exposure (bottom photo shows the user’s view)**. Credits: **(A–C)** Laboratory of Cyberpsychology, Université du Québec en Outaouais.

Historically, exposure has been accomplished *in vivo* (facing the actual stimulus or a physical representation of it; see Figure 3) and *in imago* (mental imaging of the stimulus). However, each of these techniques has major drawbacks: while a patient may be unwilling to face the actual threat *in vivo*, it might prove too difficult for a patient to mentally visualize the anxiety inducing threat. In fact, it has been reported that when patients find out that the therapy entails facing the threat, about 25% of them either refuse the therapy or terminate it (Marks, 1978, 1992; García-Palacios et al., 2001, 2007).

#### Virtual reality exposure-based therapy

Enabled by technological progress, the search for a less threatening and a more practical alternative to IVET has lead to the introduction of VRET (see Figure 3). During VRET, the patient is immersed in a VE where he faces a virtual representation of the threat. While the patients’ acceptance of such a protocol is generally higher than that of IVET (García-Palacios et al., 2001), the efficacy of the exposure-based treatment is not sacrificed. Indeed, the use of VRET has proved itself effective in treating specific phobias such as acrophobia (Emmelkamp et al., 2001, 2002; Krijn et al., 2004), arachnophobia (García-Palacios et al., 2002), aviophobia (Wiederhold, 1999; Rothbaum et al., 2000, 2002; Maltby et al., 2002; Mühlberger et al., 2003; Botella et al., 2004), claustrophobia (Botella et al., 2000), spider phobia (Michaliszyn et al., 2010), and driving phobia (Wald and Taylor, 2003; Walshe et al., 2003). In fact, a meta-analysis by Powers and Emmelkamp (2008) suggests that, in the domain of phobias and anxiety disorders, VRET is slightly, but significantly, more effective than IVET.

Virtual reality exposure-based therapy does enjoy other advantages over IVET (Botella et al., 2005). These include better control of the anxiety inducing stimulus which, of course, poses no real threat (i.e., a virtual dog can’t bite). Thus, the patient need not fear being hurt. The exposure scenarios, however complex they may be, can be stopped, paused, restarted as well as repeated, whenever and, for as many times as deemed necessary. Furthermore, the entire exposure process can be completed in the safety and privacy of the practitioner’s office. In the case of animal phobia, VRET dispenses the therapist of the problems associated with finding, taking care of, and handling live animals. Finally, some therapists find VRET more acceptable, helpful, and ethical than IVET (Richard and Gloster, 2007).

## Augmented Reality

### Distinguishing augmented reality from virtual reality

**Figure 4:**
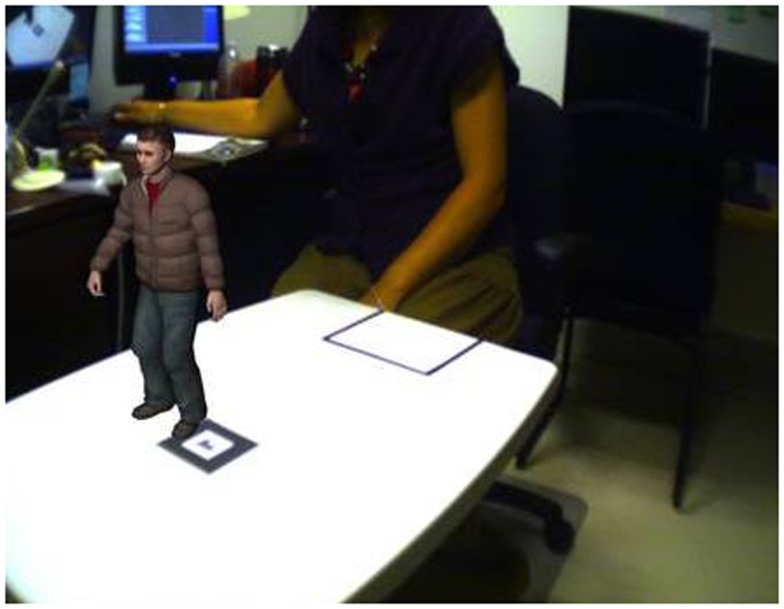
**Example of a non-synthetic environment (the researcher in the laboratory) augmented by a synthetic element (the small person standing on a non-synthetic table)**. Credit, Laboratory of Cyberpsychology, Université du Québec en Outaouais.

**Figure 5:**
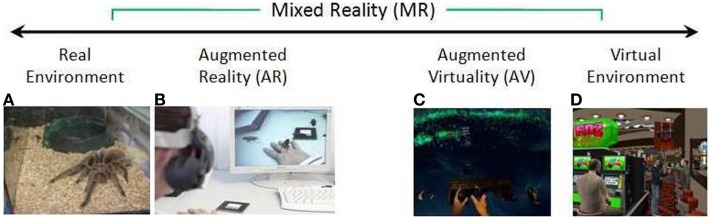
**Simplified representation of a “virtuality continuum” (Milgram and Kishino, 1994)**. Credits: **(A,B,D)** Laboratory of Cyberpsychology, Université du Québec en Outaouais **(C)** Video frame from “Vrui on Oculus Rift with Razer Hydra and Kinect,” http://www.youtube.com/watch?v=IERHs7yYsWI (Preview). Image courtesy of Oliver Kreylos.

With time, further technological advances led to the development of another method of exposure: ARET (see Figure 3). In contrast to VR systems which generate a complete VE, AR systems enhance the non-synthetic environment by introducing synthetic elements to the user’s perception of the world (see Figure 4). While VR substitutes the existing physical environment with a virtual one, AR uses virtual elements to build upon the existing environment (Azuma, 1997; Azuma et al., 2001). Milgram and Kishino (1994) present AR as a form of mixed reality (MR), that is, a “particular subclass of VR related technologies” (Milgram and Kishino, 1994, p. 1321), which, via a single display, expose the user to electronically merged synthetic and non-synthetic elements. Milgram’s Reality–Virtuality Continuum serves to illustrate where MR situates itself in comparison to real and VEs (see Figure 5). Between these two poles exist various combination levels of synthetic and non-synthetic elements: to the right of center are the environments where virtuality provides the surrounding environment (augmented virtuality), and to the left of center are the environments where reality provides the surrounding environment (AR). It is important to note that AR does not limit itself to introducing virtual elements into the physical world, it may also inhibit the perception of physical objects by overlaying them with virtual representations, such as a virtual objects or even virtual empty spaces. Although AR can be extended to hearing, touch, as well as smell (Azuma et al., 2001), this article will limit itself to the sense of vision.

In contrast to a VR user, the user of AR does not “depart” the space he occupies, thus he “maintains his sense of presence” in the non-synthetic world (Botella et al., 2005). He is, however, put in co-presence with virtual elements that are blended into the non-synthetic world. Azuma et al. (2001) propose that, to be considered AR, a system must: (1) combine real and virtual objects in a real environment; (2) run interactively, and in real time; and (3) register (align) real and virtual objects with each other. The purposes of the virtual elements include enhancing the experience and/or the knowledge of the user (Berryman, 2012). They could represent advisories (e.g., name of a building, distance to destination) or entities (e.g., an object, a person).

### About qualifying the AR experience

Thus, experiencing an AR environment is fundamentally different from experiencing a VE: unlike the user of a VE, the user of an augmented reality environment (ARE) is not “transported” to a different location, and consequently, there is no immersion *per se*. Instead, it is the virtual elements that are transported into, and aligned with, the user’s world. It could be said that in a VE, the user “intrudes” in the virtual world, while in an ARE, it is the virtual objects that “intrude” in the user’s world. Thus, the means by which the quality of a user’s experience is measured may need to be modified slightly. However, as Table 1 suggests, the instruments used to qualify a user’s experience in AR are often the same as those used in VR.

**Table 1:** **Clinical and experience related measures taken during past ARET studies**.

	Clinical measures	Experience related measures
Botella et al. (2005)	Behavior avoidance test (BAT) (adapted from Öst et al., 1991b)	Presence (two questions relating to presence)
	Degree of belief in catastrophic thought (assessed daily on scale from 0 to 100%)	Reality judgment (one question related to reality judgment)
	Fear and avoidance scales (adapted from Marks and Mathews, 1979)	
	Fear of spiders questionnaire (FSQ) (Szymanski and O’Donohue, 1995)	
	Spider phobia beliefs questionnaire (SPBQ) (adapted from Arntz et al., 1993)	
	Subjective units of discomfort scale (SUDS) (Wolpe, 1969)	
Juan et al. (2005)	Fear and avoidance scale (adapted from Marks and Mathews, 1979)	Presence (two questions relating to presence)
	Subjective units of discomfort scale (SUDS) (Wolpe, 1969)	Reality judgment (one question related to reality judgment)
Botella et al. (2010)	Behavior avoidance test (BAT) (adapted from Öst et al., 1991b)	N/A
	Degree of belief in catastrophic thought (assessed on a 10-point Likert scale)	
	Fear of spiders questionnaire (FSQ) (Szymanski and O’Donohue, 1995)	
	Spider phobia beliefs questionnaire (SPBQ) (adapted from Arntz et al., 1993)	
	Subjective units of discomfort scale (SUDS) (Wolpe, 1969)	
	Target behaviors (adapted from Marks and Mathews, 1979)	
Bretón-López et al. (2010)	Subjective units of discomfort scale (SUDS) (Wolpe, 1969)	Presence (two items from presence and reality judgment questionnaire; Baños et al., 2005)
		Reality judgment (assessed on a 10-point scale)
Wrzesien et al. (2011a)	Anxiety (assessed on a 10-point Likert scale)	N/A
	Avoidance (assessed on a 10-point Likert scale)	
	Behavioral avoidance test (assessed on a 13-point Likert scale)	
	Belief in catastrophic thoughts (assessed on a 10-point Likert scale)	
Wrzesien et al. (2011b)	Anxiety (assessed on a 10-point Likert scale)	N/A
	Avoidance (assessed on a 10-point Likert scale)	
	Behavioral avoidance test (BAT) (adapted from Öst, 2000)	
	Belief in catastrophic thoughts (assessed on a 10-point Likert scale)	
Wrzesien et al. (2013)	Self efficacy belief (assessed on a seven-point scale)	Presence and reality judgment questionnaire (assessed on 10-point scales)
	Spiders and cockroach anxiety and avoidance questionnaire (assessed on a seven-point scale)	
	Subjective units of discomfort scale (SUDS) (assessed on a 10-point scale)	

In an ARE, measures of realism (degree of convergence between the expectations of the user and the actual experience in the VE) and reality (level to which the user experiences the hybrid environment as authentic) are still pertinent. However, this may not be the case for presence. If Heeter’s (1992) conceptualization of presence is used as reference, it can be argued that measures of the environmental presence (the feeling that the environment seems to acknowledge the user’s movements by reacting to his actions; Heeter, 1992) and social presence (the feeling of not being alone in the environment; Heeter, 1992) can also be pertinent to qualify the experience of the user in an ARE. However, unlike VR where social presence measures the level of “togetherness” between the user and virtual agents, in AR, the level of “togetherness” between the user and individuals physically present in the environment may also be of interest. On the other hand, a measure of personal presence does not seem pertinent in an ARE; indeed, the user is not “transported” to a different location, and thus, the value of measuring the level of personal presence in a location the user never left may be questionable.

On the other hand, a measure addressing the alignment of real and virtual elements could contribute to an overall assessment of the quality of a user’s experience in an ARE; this could be in the form of a measure of co-existence between the virtual and the non-virtual elements. Further co-existence measures could assist in qualifying the experience of an ARE. These could include co-existence measures between the user and virtual elements, as well as between the user and non-virtual elements (this last measure could be used as a baseline to put the level of co-existence between user and non-virtual elements into context).

#### History of augmented reality

The term “augmented reality” was introduced in 1990 by Tom Caudell while working on Boeing’s Computer Services’ Adaptive Neural Systems Research and Development project in Seattle (Carmigniani et al., 2011). There, alongside David Mizell, he developed an application that displayed a plane’s schematics on the factory floor (Vaughan-Nichols, 2009), thereby saving the mechanics the difficult task of interpreting abstract diagrams in manuals (Berryman, 2012). Two further AR pioneering projects were Rosenberg’s Virtual Fixtures and Feiner and colleagues’ knowledge-based augmented reality for maintenance assistance (KARMA). Results of the Virtual Fixtures project suggested that teleoperator performance can be enhanced by overlaying abstract sensory information in the form of virtual fixtures on top of sensory feedback from a remote environment (Rosenberg, 1993). KARMA used 3D graphics to guide a user through the steps to carry out some of the complex tasks of printer maintenance/repair (Feiner et al., 1993). In 1993, Loral Western Development Laboratories took AR to a new level by introducing AR to live training involving combat vehicles (Barilleaux, 1999), and in 1994, in a completely different field, Julie Martin created “Dancing in Cyberspace,” the first AR theater production featuring dancers and acrobats interacting with virtual object in real time (Cathy, 2011).

Some of the other important developments for AR include: Kato and Billinghurst (1999) created AR Toolkit, the first widely held software to solve tracking and object interaction; the next year, Thomas et al. (2000) developed ARQuake, the first outdoor mobile AR video game; the year 2008 saw the development of applications such as Wikitude, which uses a smartphone’s camera view, internet, and GPS (or Wifi) positioning to display information about the user’s surroundings (Perry, 2008); in 2009, AR Toolkit was brought to the web browser by Saqoosha (Cameron, 2010), and SiteLens, an application that allows visualization of relevant virtual data directly in the context of the physical site, was introduced (White and Feiner, 2009); in 2011, Laster Technologies incorporated AR in ski goggles (e.g., ITR News, 2011), while Total Immersion created D’Fusion, a platform to design AR projects for mobile, web based, and professional applications (Maurugeon, 2011); and finally, in 2013, Google began to test Google Glass, a pair of AR glasses connected wirelessly to the internet via the user’s cellphone wireless service.

While the coining of the phrase “augmented reality” is an important historical reference, the concept at the source of the phrase had made its mark long before 1990. In fact, it was in 1901 that Lyman Frank Baum, an American author of children’s books, put on paper what may have been the first idea for an AR application. In his novel titled *The Master Key* (1901), he wrote:

“The third and last gift of the present series,” resumed the Demon, “is one no less curious than the Record of Events, although it has an entirely different value. It is a Character Marker.” “What’s that?” inquired Rob.

“I will explain. Perhaps you know that your fellow-creatures are more or less hypocritical. That is, they try to appear good when they are not, and wise when in reality they are foolish. They tell you they are friendly when they positively hate you, and try to make you believe they are kind when their natures are cruel. This hypocrisy seems to be a human failing. One of your writers has said, with truth that among civilized people things is seldom what they seem.”

“I’ve heard that,” remarked Rob.

“On the other hand,” continued the Demon, “some people with fierce countenances are kindly by nature, and many who appear to be evil are in reality honorable and trustworthy. Therefore, that you may judge all your fellow-creatures truly, and know upon whom to depend, I give you the Character Marker. It consists of this pair of spectacles. While you wear them every one you meet will be marked upon the forehead with a letter indicating his or her character. The good will bear the letter “G,” the evil the letter “E.” The wise will be marked with a “W” and the foolish with an “F.” The kind will show a “K” upon their foreheads and the cruel a letter “C.” Thus you may determine by a single look the true natures of all those you encounter.” (Baum, 1901, pp. 37–38)

Although potentially useful, the “character marker” was not developed into a concrete application. However, around that same timeframe, an AR-like application saw the light of day: the reflector (“reflex”) gunsight. The concept behind the gunsight was published by Grubb (1901): “the sight which forms the subject of this paper attains a similar result not by projecting an actual spot of light or an image on the object but by projecting what is called in optical language a virtual image upon it” (Grubb, 1901, p. 324). Although, its first employment is difficult to date exactly, the reflector gunsight was operational in German fighter aircraft by 1918 (Clarke, 1994). Installed in front of the pilot, and in line with the aircraft’s gun(s), the reflector gunsight consisted of a 45° angle glass beam splitter on which an image (e.g., an aiming reticle) was projected (Clarke, 1994); thus, it superposed virtual elements on real world elements. Its purpose was to assist pilots in hitting their targets by providing them with a reference aiming point. However, according to Azuma and colleagues’ definition, this type of concept cannot be considered an AR system. Indeed, it only met two of their three criteria of AR: it combines real and virtual objects in a real environment and it runs interactively, and in real time; it does not, however, align real and virtual objects with each other. On the other hand, the reflector gunsight does meet Berryman’s stated purpose of AR: the enhancement of the experience and/or the knowledge of the user (Berryman, 2012).

The difficulty of categorizing this type of application as AR persists for the follow-on systems. As it was known that the trajectory of the bullets was influenced by the shooting aircraft’s flight parameters, the newer generation of gunsights started to take these into account when displaying the aiming reticle. This type of application does, to a certain extent, take real world parameters into consideration, but it still does not align real and virtual objects. Thus, it does not quite meet Azuma and colleagues’ definition of AR.

The first operational system that did meet all three of Azuma and colleagues’ premises of AR seems to have been the AI Mk VIII Projector System (earlier variants had been successfully tested but never entered service). As the name suggests, the radar picture of the AI Mk VIII radar picture was projected onto the pilot’s windscreen, thereby superimposing the virtual cue onto the real world position of the target aircraft (as seen from the cockpit; Clarke, 1994). Although the alignment of virtual and real elements may have been somewhat rudimentary, the AI Mk VIII seems to have been, long before the term “augmented reality” was coined, the first AR system.

**Figure 6:**
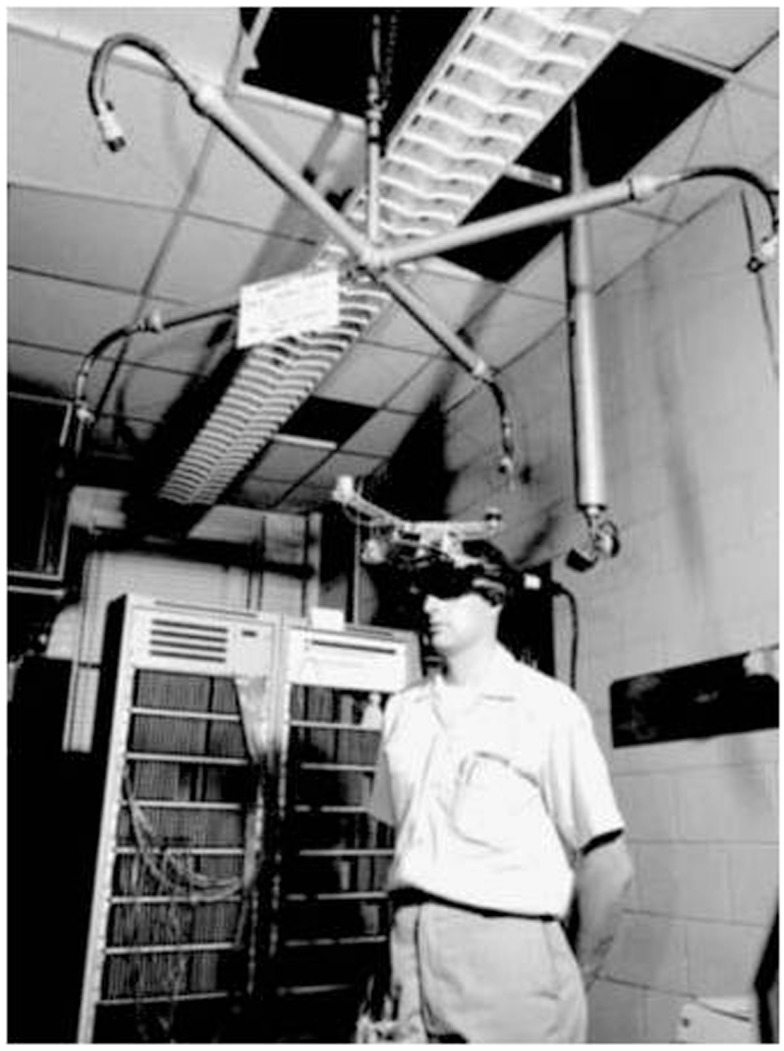
**The Sword of Damocles (circa 1968)**. Reprinted from Sherman and Craig (2003), with permission from Elsevier.

The concept of projecting flight parameters and target information on a see-through surface eventually led to the developments of the head-up display (HUD), the helmet mounted sight (HMS), and the helmet mounted display (HMD). The latter, invented in the 1960s by Professor Ivan Sutherland and his graduate student Bob Sproull, can be considered as one of the major technological breakthrough that furthered the development of AR (and VR; Berryman, 2012). Nicknamed the “The Sword of Damocles” (see Figure 6) due to the fact it was suspended from the ceiling over the user, their see-through head mounted display was able to present simple 3D wireframe models of generated environments (Sutherland, 1968). In the 1970s and 1980s, the United States Air Force and the National Aeronautics and Space Administration were among the organizations that further researched AR and its potential applications (Feiner, 2002). The integration of HMSs (in the 1970s), and HMDs (in the1980s) in fighter aircraft were among the concrete results of this research; today’s Google Glasses can be seen as a technological offspring of the HMD. Although military applications may have been an important motor in the development of AR technologies, entertainment oriented applications, such as Myron Krueger’s Videoplace, also occupy important places in the history of AR (Dinkla, 1997).

### About the definition of AR

Thus, AR applications had been around long before a term was assigned to the concept. However, a look back at history and forward to the future of AR also reveals that the present definition of AR excludes some AR-like functionalities, such as the display of information that is overlaid onto, but not merged with, the real world (e.g., speed of car projected onto the inside of the windshield). As AR functionalities may co-exist with such AR-like functionalities (e.g., an arrow to indicate where to turn and an indication of the distance to go before that turn), it could be useful to find a term that describes functionalities that don’t register real and virtual objects with each other. Using Azuma and colleagues’ widely accepted definition of AR as an anchor, the authors of the present paper propose the term “non-registered augmented reality” (NRAR) to describe functionalities that: (1) combine real and virtual objects in a real environment; and (2) run interactively, and in real time; but (3) don’t register (align) real and virtual objects with each other.

### Enabling technologies

**Figure 7:**
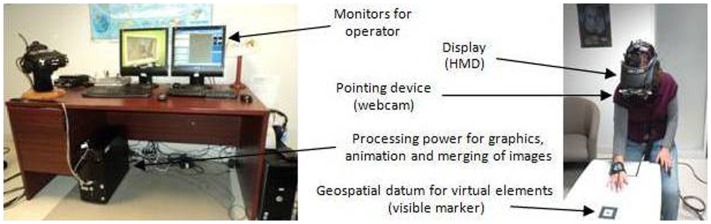
**Example of an AR set-up**. Credit, Laboratory of Cyberpsychology, Université du Québec en Outaouais.

While the exact configurations of AR systems vary, their common elements include: (a) a means of providing a geospatial datum to the synthetic elements; (b) a surface to project the environment to the user; (c) sufficient processing power to generate the 3-D synthetic elements and merge them with the pointing device’s input; and (d) adequate graphics power to animate the scene on the display (see Figure 7). A detailed review of each of the hardware pieces of AR systems is beyond the scope of this paper (for a more detailed overview of AR technologies, see Carmigniani et al., 2011), but it is worthwhile to mention some details about possible methods of geospatial referencing and the types of visual displays.

In order to achieve a near seamless integration of the virtual elements in the non-synthetic environment, 3D tracking must be able to define accurately the orientation and position of the user relative to the scene. To this end, magnetic, mechanical, acoustic, inertial, optical, or hybrid technologies have been used (Bowman et al., 2005). These technologies may provide: (a) the user’s and the virtual elements’ respective positions and orientations on a geospatial grid (e.g., GPS); or (b) the position and orientation of the user relative to a reference point recognizable by the pointing device (e.g., a visual marker). In the case of markers, these may be visible or invisible to the human eye.

The visual displays used in AR may be categorized as projective, handheld, and head-level devices (Azuma et al., 2001). The latter can be as bulky as head mounted displays, as light as eyeglasses, and as inconspicuous as contact lenses. Two types of systems may display the composite environment to the user: (a) a video see-through (VST) AR system (Botella et al., 2005; Juan et al., 2005); and (b) an optical see-through (OST) system (Juan et al., 2007). While a VST system exposes the user to images composed of a video feed of the non-synthetic environment merged with synthetic elements, an OST system overlays the synthetic elements on a transparent surface (e.g., glass) through which the user sees the non-synthetic environment. It is worthwhile to note that, unlike an OST system, a VST system requires a means to capture the non-synthetic environment (e.g., a web cam). In terms of user experience, a major difference between these two systems is the effect of computer graphics latency. Indeed, the user of an OST system may detect a lack of synchronization between the environment (observed in real time) and the view of the synthetic elements (displayed after some degree of graphics latency). A VST system, on the other hand, can delay the display of the video feed to synchronize video and graphics; as a result, the user detects no delay between the video of the physical world and the virtual elements, but may detect a delay between actual head movement and the head movement shown in the video of the physical world. Thus, in choosing the appropriate AR system for a particular application, one of the choices to be made is whether it is less adverse to have: (a) a slight lack of synchronicity between the environment and the synthetic elements (as in an OST); or (b) the graphics latency applied to the video transmission of the environment, thus creating a very slight time lag between actual movement felt by the body and movement detected by the user’s vision (as in a VST).

### Technical challenges

One of the important technical challenges of AR is to make the integration of the virtual elements into the non-synthetic environment as seamless as possible, thus giving the user the illusion of the co-existence of virtual and non-synthetic elements in a “unique world” (Botella et al., 2010, p. 402). The illusion must be maintained during the entire exposure, regardless of the angle or height from which the user observes the virtual elements. This requirement of complete fusion of the virtual elements into the non-synthetic world implies significant programing, and this challenge is further accentuated if the virtual elements are not stationary (e.g., a group of virtual spiders moving about a non-virtual table).

### Augmented reality applications

As the technology supporting AR developed, AR has been researched and used in various fields such as education (Kerawalla et al., 2006; Arvanitis et al., 2009), medicine (De Buck et al., 2005), architecture (Grasset et al., 2001), maintenance (Schwald and Laval, 2003), entertainment (Özbek et al., 2004), and disaster management (Leebmann, 2006). In the field of mental health, the use of new technologies holds many promises (e.g., Botella et al., 2010). Like VR, AR allows patients to have easier access to mental health services and, due to the strong representational and immersion capability of these technologies, AR can enhance the patients’ engagement in the treatments (Coyle et al., 2007).

#### Augmented reality in exposure-based therapy against phobia

##### Advantages of ARET over VRET

**Figure 8:**
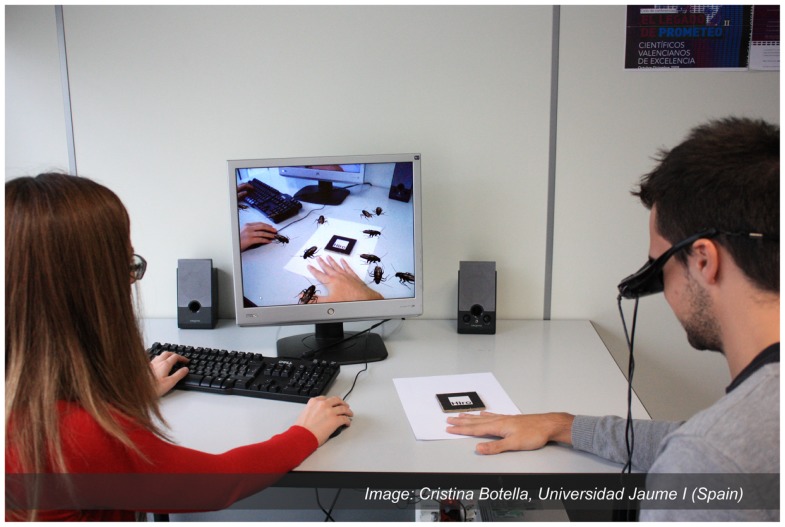
**Example of an ARET environment**. Copyright. Labpsitec. Universitat Jaume I. Spain.

In the treatment of phobias via exposure-based treatments, ARET enjoys the same advantages over IVET as VRET does (e.g., control over the scenario, safety, variety of stimuli, confidentiality, repetition, and self-training). However, as AR requires that only a few virtual elements be designed, the cost of producing the environment is reduced. Furthermore, unlike VR, AR does not “extract” the user from the real world (Dünser et al., 2011). Thus, the AR user’s experience of the environment does not hinge on his ability to “build” a sense of presence. Furthermore, the user of an ARE is able to see his own body interact with the virtual elements (versus seeing a virtual representation of his body; see Figure 8). By embedding the virtual fear element in the real environment and allowing a direct “own-body” perception of that environment, the ecological validity of the scenario is increased (Dünser et al., 2011). The implications of these advantages of AR over VR include a less costly system that could elicit greater sense of presence and better reality judgment of the objects (Botella et al., 2005).

##### Efficacy of ARET in treating small animal phobia

Botella et al. (2005) seem to have published the first study regarding the treatment of a specific phobia using ARET. In a single subject study applying the “one-session treatment” guidelines of Öst et al. (1991b), they successfully treated a participant that initially met the DSM-IV (American Psychiatric Association, 2000) diagnosis of small animal phobia (in this case, cockroaches). During the course of the study, they demonstrated not only the ability of the virtual cockroaches to activate a patient’s anxiety, but also a reduction in anxiety as the 1-h period of exposure progressed. More specifically, important decreases in the scores of fear, avoidance, and belief in catastrophic thought were measured (the types of measures are shown at Table 1). Furthermore, after the treatment, the participant was capable of approaching, interacting, and killing live cockroaches. The results were maintained in a follow-up conducted 1 month after the termination of the treatment. Although this study showed promising results, the authors remark that they needed to be confirmed with bigger samples and other pathologies (Botella et al., 2005).

That same year, Juan et al. (2005) published a similar study involving nine participants that met the DSM-IV-TR’s (American Psychiatric Association, 2000) criteria for a specific phobia (five participants feared cockroaches and four feared spiders). Using the “one-session treatment” guidelines developed by Öst et al. (1991b), the ARET protocol followed four distinct steps: (a) simple exposure to a progressively increasing number of animals (cockroaches or spiders, as applicable); (b) approaching a progressively increasing number of the animals with the hand; (c) looking under four boxes to uncover, or not, the feared animal(s); and (d) observing the therapist repeatedly crush spiders or cockroaches and throw them into a box, before doing so oneself. The study demonstrated that the AR system was able to induce anxiety in individuals suffering from spider or cockroach phobia. In all cases, the treatment successfully reduced the participants’ fear and avoidance of the target animal (the types of measures are shown at Table 1). In fact, after the treatment, all of the participants were able to approach the live animals, interact with them, and kill them by themselves. The authors point out that their results are positive for the future of AR in psychology, but that follow-on studies should include a larger sample and a control group.

In 2010, Botella and colleagues published the results of another study testing an AR system for the treatment of cockroach phobia (Botella et al., 2010). Compared to the previous studies on ARET, this one introduced a longer period of post-treatment retest (3, 6, and 12 months). The six participants met the DSM-IV-TR (American Psychiatric Association, 2000) criteria for Specific Phobia animal type (Cockroach Phobia), and the treatment was preceded by two 60 min assessment periods during which: (a) the ADIS-IV for specific phobia was administered; (b) the target behaviors as well as the exposure hierarchy were established; and (c) the participants completed other self-report measures. The intensive exposure-based treatment, lasting up to 3 h, followed the “one-session treatment” guidelines developed by Öst et al. (1991b). Various measures of anxiety, avoidance and beliefs in negative beliefs were taken pre-, per-, and post-treatment (the types of measures are shown at Table 1). The data collected in this study indicate that the AR system was able to induce anxiety in all participants. Post-treatment, all of the patients: (a) had improved significantly in the level fear, avoidance and belief in negative thoughts related to the main target behavior (the gains were maintained at 3, 6, and 12-month follow-up periods); and (b) were able to interact with real cockroaches (an act they were unable to carry out pre-treatment). Thus, the results of this study support the finding of the aforementioned ones, that is, ARET can be efficacious against a specific animal phobia. However, the authors point to some of the limitations of their study, namely, the small number of participants, the absence of a control group, and the absence of a formal test for cybersickness; the latter refers to a form of motion sickness that can be experienced by the user of an immersive synthetic environment.

That same year, Breton-Lopez and colleagues published a study aiming to explore the ability of an AR system to induced anxiety in six participants diagnosed with the DSM-IV-TR’s (American Psychiatric Association, 2000) criteria of cockroach phobia. As the secondary objective, the authors aimed to verify their system’s ability to elicit a sense of presence and reality judgment. In the ARE, the participants were exposed, in an order established to each individual’s hierarchy of fears, to various elements programed in the AR system. Throughout this process, the participants rated their levels of anxiety, presence, and reality judgment (the types of measures are shown at Table 1). Regarding the level of anxiety, the results confirmed that the system is capable of inducing anxiety in all participants, and that the levels of anxiety decreased progressively during a prolonged exposure to the anxiety inducing stimuli. The novel aspect of the findings is that the exposures to “one insect in movement” and “more insects in movement” elicited, in all participants, higher levels of anxiety than stationary insects. This result suggests that the movement of the animal may be an important element to integrate in this type of application. Regarding presence, the authors report that all participants were able to “immerse themselves” in the AR environment and that they attributed a high level of reality to the cockroaches. Overall, the authors conclude that their results confirm the ability of their AR system to contribute to the treatment of cockroach phobia.

In 2011, Wrzesien and colleagues evaluated the Human Computer Interface and clinical aspects of their AR system for cockroach phobia (Wrzesien et al., 2011b). To this end, five “clients” (neither the diagnostic nor the instrument used for the diagnosis is reported) were treated through individual one-session (Öst, 2000) ARET clinical guidelines. The data collected showed post-treatment improvements in the levels of anxiety, avoidance, and belief in catastrophic thoughts (the types of measures are shown at Table 1). More specifically, while the clients had not been able to get closer than 1 or 2 m to a real cockroach prior to the treatment, after the therapy, they were able to put a hand into a terrarium with a real cockroach. The authors conclude that, although the ARET system was effective in these clinical cases, the small size of the sample and the absence of a control group should be improved to confirm the results.

That same year, Wrzesien and colleagues published what seems to be the first (preliminary) results concerning a comparative study between IVET and ARET (Wrzesien et al., 2011a). For the purpose of this study, 12 participants that met the DSM-IV-TR (American Psychiatric Association, 2000) criteria for a specific phobia to small animals (spiders and cockroaches) were randomly assigned to an IVET or an ARET group. The therapeutic sessions, which followed the “one-session treatment” protocol (Öst, 2000), included a single intensive exposure session of up to 3 h; the exposure exercises had been defined previously and were ordered according to each participant’s hierarchy of fears. Measures of avoidance, anxiety, and irrational thoughts were taken throughout the protocol (the types of measures are shown at Table 1). While the results of this pilot study suggest that both ARET and IVET are clinically effective, some differences were noted between the groups. For both groups, the clinical measures of anxiety, avoidance, and avoidance behavior decreased significantly after the therapeutic session. However, the clinical measure of belief in catastrophic thought only improved significantly in the ARET group. Between the groups, the authors report a significantly higher improvement of the avoidance score of the IVET group, but no improvement differences in either, the anxiety, the belief in catastrophic thought or the behavior avoidance measures. The authors suggest that the small size of the clinical sample may have played a role in the differences between the groups.

Botella et al. (2011) published the results of another single case study combining a serious game on a mobile phone with ARET. As this study involved AR in the treatment of a phobia (in this case, cockroach phobia), it is mentioned here. However, the combination of protocols goes beyond the scope of this paper; thus, this study is not reviewed.

In 2013, Wrzesien and colleagues tested a new display technology they called therapeutic lamp (TL), a projection-based AR system for therapy for small-animal phobia (Wrzesien et al., 2013). Unlike the head-level AR systems, their system has the advantage of not requiring the use of a head mounted display. The non-clinical sample of 26 volunteers underwent a single exposure-based therapy protocol comprised of 12 exercises (from least to most anxiety inducing). The results indicated that anxiety scores, although relatively high at the beginning of each exercise, dropped by the end and after the session. Furthermore, the participants’ belief in their capacity to face a cockroach had increased significantly after the session (the types of measures are shown at Table 1). The authors conclude that TL can be a useful therapeutic tool for other psychological disorders, but that their results need to be validated with phobia patients.

##### About the types of phobias treated by ARET

All of the cases of ARET research projects found in preparation for this paper involved small animal phobia. One of the factors behind this restricted use of ARET may be related to the use of visual markers to track the orientation and position of the user relative to the scene. Indeed, the use of visual markers implies that as soon as part of the marker is not in the user’s field of view, the virtual stimulus disappears completely. This technological limitation prevents the use of ARET in treating certain phobias (e.g., larger animal phobias, the fear of public speaking, the fear of thunder and lightning). While it may be difficult for cyberpsychology laboratories working without significant technical support to implement alternative tracking technologies, it could be constructive for those who do benefit from such support to experiment ARET protocols using alternative tracking methods (for examples, refer to the section titled “enabling technologies”); such developments may unlock ARET’s access to many potentially useful treatment protocols. One of these may be the treatment of social phobia. Presently, some of the VEs destined to provide support in the treatment of social phobia rely on public speaking tasks. Depending on the target population, the environment often consists of public speaking rooms such as auditoriums, conference rooms, and classrooms. In this type of situation, one advantage of ARET is that the exposure could take place in the actual places the patient encounters his difficulties (e.g., an accounting officer who finds it difficult to present the financial results to the board members, or a child who isn’t able to present in class). Thus, assuming that the required tracking technology is developed, AR could provide such *in situ* training.

## Conclusion

The purpose of this paper was to review the move from VRET to ARET. Unlike VR, which entails a complete VE, AR limits itself to producing certain virtual elements to then merge them into the view of the physical world. Although the general public may only have become aware of AR in the last few years, AR type applications have been around since beginning of the twentieth century. Since then, technological developments have enabled an ever increasing level of seamless integration of virtual and physical elements into one view. Like VR, AR allows the exposure to stimuli which, due to various reasons, may not be suitable for real-life scenarios. As such, AR has proven itself to be a medium through which individuals suffering from specific phobia can be exposed “safely” to the object(s) of their fear, without the costs associated with programing complete VEs. Thus, ARET can offer an efficacious alternative to some less advantageous exposure-based therapies. Above and beyond presenting what has been accomplished in ARET, this paper also raised some AR related issues, and proposes potential avenues to be followed. These include the definition of an AR related term, the type of measures to be used to qualify the experience of ARE users, as well as the development of alternative geospatial referencing systems, which themselves, may open the door to other ARET applications, such as the treatment of social phobia. Overall, it may be said that the use of ARET, although promising, is still in its infancy but that, given a continued cooperation between clinical and technical teams, ARET has the potential of going well beyond the treatment of small animal phobia.

## Conflict of Interest Statement

The authors declare that the research was conducted in the absence of any commercial or financial relationships that could be construed as a potential conflict of interest.
